# Immune interactions and regulation with CD39^+^ extracellular vesicles from platelet concentrates

**DOI:** 10.3389/fimmu.2024.1397967

**Published:** 2024-06-14

**Authors:** Adèle Silane Delorme, Alexandra Laguide, Marie Tamagne, Marion Klea Pinheiro, Léonie Cagnet, Deborah Neyrinck-Leglantier, Mehdi Khelfa, Sabine Cleophax, France Pirenne, Benoît Vingert

**Affiliations:** ^1^ Univ Paris Est Creteil, Institut National de la Santé et de la Recherche Médicale (INSERM), Institut Mondor de la Recherche Biomédicale (IMRB), Creteil, France; ^2^ Etablissement Français du Sang, Ivry sur Seine, France; ^3^ Laboratory of Excellence, Biogénèse et Pathologies du Globule Rouge (GR-Ex), Paris, France

**Keywords:** immune activation, extracellular vesicles, alloimmunization, cellular microparticles, immunoregulatory molecules, CD39 ectoenzyme, transfusion

## Abstract

**Introduction:**

CD39 plays an important role in the immunoregulation and inhibition of effector cells. It is expressed on immune cells, including Tregs, and on extracellular vesicles (EVs) budding from the plasma membrane. Platelet transfusion may induce alloimmunization against HLA-I antigens, leading to refractoriness to platelet transfusion with severe consequences for patients. Tregs may play a key role in determining whether alloimmunization occurs in patients with hematologic disorders. We hypothesized that CD39^+^ EVs might play an immunoregulatory role, particularly in the context of platelet transfusions in patients with hematologic disorders. Such alloimmunization leads to the production of alloantibodies and is sensitive to the regulatory action of CD39.

**Methods:**

We characterized CD39^+^ EVs in platelet concentrates by flow cytometry. The absolute numbers and cellular origins of CD39^+^ EVs were evaluated. We also performed functional tests to evaluate interactions with immune cells and their functions.

**Results:**

We found that CD39^+^ EVs from platelet concentrates had an inhibitory phenotype that could be transferred to the immune cells with which they interacted: CD4^+^ and CD8^+^ T lymphocytes (TLs), dendritic cells, monocytes, and B lymphocytes (BLs). Moreover, the concentration of CD39^+^ EVs in platelet concentrates varied and was very high in 10% of concentrates. The number of these EVs present was determinant for EV-cell interactions. Finally, functional interactions were observed with BLs, CD4^+^ TLs and CD39^+^ EVs for immunoglobulin production and lymphoproliferation, with potential implications for the immunological management of patients.

## Introduction

Hematologic disorders and their treatment can lead to thrombocytopenia, which can be managed by polytransfusion with platelet concentrates (PCs). Such transfusions may activate the immune system of the patient (1, 2), potentially leading to alloimmunization against HLA-I, diagnosed principally on the basis of alloantibody production, although cell-specific responses have also been detected in patients with hematologic disorders (2). Immunologically, this raises the question as to whether anti-HLA responses occur in patients who develop immune responses, including anti-tumor immune responses, more easily.

However, some patients never develop an anti-HLA response. This lack of anti-HLA response may be to the result of an immunoregulation, and dependent on a transfusion-related element, then it is important to identify element concerned to improve the evaluation of transfusion products.

The difference in immunoregulation between patients may also be explained by the content of the platelet concentrates used, including their levels of extracellular vesicles (EVs). EVs are heterogeneous in size and cellular origin. They are known to have immunoactive properties. The immunoactive properties of two types of EVs have been explored: exosomes, which are small (mean diameter of 40–100 nm) and derived from intracellular membrane compartments, and larger extracellular vesicles (160–900 nm) also known as microparticles (MPs) or ectosomes, which bud from the plasma membrane of cells (3, 4). We focus here exclusively on MPs, as defined by the International Society for Extracellular Vesicles (5–7).

Extracellular MPs are naturally present in the blood and in blood products (4, 8, 9). They are formed by budding from the cell membrane, resulting in the expression on EVs of large numbers of diverse membrane proteins (e.g. cell origin and activation markers), cytoplasmic molecules, such as cytokines, and genetic material (9).

The precise roles of extracellular MPs in immunoregulatory functions remain unclear, largely due to technical differences between studies, including the absence of extracellular MP purification by flow cytometry. However, these EVs clearly have many immunomodulatory effects on diverse immune cells, and are, therefore, an integral part of the immune system (4, 9–15). Our studies on EVs purified by flow cytometry have confirmed these effects (8, 10–12, 16).

In the context of transfusion, there is growing interest for the role of EVs derived from transfused platelet compounds on immune system cells. This is particularly the case in lymphoma patients who are candidates for polytransfusion and who present basal immune disturbances related to the development of their tumor. EVs can amplify the inhibition of the anti-tumor response in lymphoma patients or, on the contrary, help to induce alloimmunization. Indeed, the EVs present in the transfused blood products may express immune checkpoint molecules, including CD39 in particular (10). CD39 has been primarily described as a Treg marker and plays an important role in immunoregulation (17). Together with CD73, CD39 acts as an ectoenzyme in a cascade leading to the conversion of ATP into adenosine diphosphate and cyclic adenosine monophosphate, resulting in the generation of extracellular adenosine. This extracellular adenosine has immunosuppressive properties (18–20). CD39 may also be expressed on neutrophils, monocytes, B lymphocytes (BLs), natural killer cells (NKs), conventional CD4^+^ TLs, Th17 cells, and CD8^+^ TLs (21–23).

High levels of CD39 expression have been observed in various solid tumors and lymphomas (21, 24, 25) and at the surface of EVs, although generally either exclusively on exosomes or without distinction between exosomes and MPs (26, 27). This cellular expression of CD39 is particularly important in patients with acute myeloid leukemia (AML), whose immune responses to AML can be predicted from the composition of their Treg subsets (28). We have also recently demonstrated an important role for platelet transfusion and alloimmunization status in the phenotype and immunosuppressive functions of Tregs from AML patients (1). Like CD73^+^ EVs, these CD39^+^ EVs may participate in immunomodulation (27, 29, 30).

We hypothesized that CD39^+^ EVs might have immunoregulatory effects, particularly in the context of platelet transfusion for patients with hematologic disorders. The objectives of this study were to determine the cellular origin and the phenotype of CD39^+^ EVs from PCs by evaluating the presence, at the surface of the EVs, of immunoregulatory molecules, most of which were reported in previous studies: TGFβ, LAG3 (CD223), CD40L (CD154), PDL1 (CD274), PD1 (CD279), CTLA4 (CD152), TIM3 (CD366), FASL (CD178) plus PDL2 (CD273), BTLA (CD272), and CD73 (10). Of course, it is also possible that EVs express molecules involved in cell activation. Indeed, we previously showed that TGFβ^+^ EVs could facilitate lymphoproliferation (10). We therefore measured the expression of CD80 and OX40L(CD252) on CD39^+^ EVs. These molecules are present at the surface of antigen-presenting cells (APCs) and are crucial for the synapses between APC and T cells and, thus, ultimately, for the activation of T and B lymphocytes (31).

We also investigated the interactions between CD39^+^ EVs and CD4^+^ TLs, CD8^+^ TLs, dendritic cells (DCs), monocytes, and BLs. The mechanisms of interaction between cells and EVs remain incompletely understood but may lead to the presence of immune ligands/receptors on the surface of EVs (13, 32). We recently showed that the CD27 (a member of the tumor necrosis factor receptor superfamily) and CD70 expressed on EVs can be transferred onto CD4^+^ TLs, thereby increasing cell activation (12). In the absence of a known membrane ligand for CD39, the co-expression of immunomodulatory molecules on the surface of CD39^+^ EVs also enabled us to assess the specific mechanisms of interaction.

Finally, given their role in alloimmunization, we also investigated the impact of CD39^+^ EVs on the functionality of CD4^+^ TLs and BLs in the humoral response. The CD39-expressing EVs studied were all collected from samples taken from PCs distributed to polytransfused patients treated for hematologic malignancies. The study of cell-EV interactions was made possible by an original technique described elsewhere (11, 12) in which cells and EVs are labeled separately with antibodies directed against proteins of interest conjugated to different fluorochromes, such that the type of fluorescence observed indicates the origin of the protein. Interactions were studied by flow cytometry, distinguishing between the cells with and without expression of the fluorochrome labeling the EVs. For studies of the function of CD39-expressing EVs, these vesicles were purified by flow cytometry (10–12).

Many interactions occur between these EVs and immune system cells, depending on the number of EVs present and the expression of other molecules present on the surface of these CD39^+^ EVs. The interactions between BLs, CD4^+^ TLs and CD39^+^ EVs were functional for immunoglobulin production and lymphoproliferation, with potential implications for the immunological management of patients.

## Materials and methods

### Biological samples

Fresh blood samples were collected from healthy donors (HDs) for PBMC isolation. These samples, collected into sodium heparin-containing tubes (BD Biosciences, Franklin Lakes, NJ), were provided by the French national blood bank (*Etablissement Français du Sang*, EFS).

For all assays, the EVs studied were obtained from samples of the PCs delivered to polytransfused patients. Thus, EVs were isolated from fresh (less than 24 hours old) HD platelet concentrates sampled at the platelet preparation laboratory of the EFS. The platelet concentrates used were either apheresis platelet concentrates (aPCs) or pooled platelet concentrates (pPCs). None of the HDs had suffered an infection (bacterial, viral, fungal, or yeast) or been vaccinated in the 30 days preceding inclusion, and none had received a platelet transfusion. All the participants gave written informed consent.

### EV-enriched preparation

EVs were isolated as previously described (8, 10–12, 16). Briefly, enrichment in EVs was achieved by differential centrifugation of the PCs at an initial speed of 3,000 x *g* (4°C, 10 minutes), with the supernatant then centrifuged at 13,000 x *g* (4°C, 10 minutes) to prepare a “platelet-free supernatant”. A hematology analyzer was used to check for the absence of platelets in EV preparations (ABX Micro ES 60, Horiba, Kyoto, Japan). No platelet budding was ever detected at this first stage (**Supplementary Figure 1**). EVs were concentrated from the “platelet-free supernatant” by centrifuging this supernatant for 1 hour at 100,000 x *g* (4°C). The resulting pellet, the “EV concentrate”, containing the EVs was resuspended in filter-sterilized PBS (passage through a filter with 0.1 µm pores) for flow cytometry.

### EV phenotyping

EVs from the EV-enriched preparation were labeled as previously described (8, 10–12, 16), with fluorochrome-conjugated monoclonal antibodies. Fluorescence was assessed with a 20-parameter LSR Fortessa flow cytometer with a small-particle option (BD Biosciences) based on photomultiplier (PMT)-coupled forward scatter (FSC) detection. This mode of detection was used to ensure the optimal detection of EVs with diameters of 160 to 900 nm. The performance of the flow cytometer was checked before each assay with CS&T beads. Megamix-Plus FSC and SSC beads (BioCytex, Marseille, France) of known dimensions (beads with diameters ranging from 160 nm to 900 nm: 160 nm, 200 nm, 240 nm, 300 nm, 500 nm and 900 nm) were used to standardize the FSC-PMT parameters and to define the EV gate. The sensitivity of vesicle detection was checked with other latex (Spherotech beads, Lake Forest, IL) and silica (ApogeeFlow beads, Hertfordshire, United Kingdom) beads (**Supplementary Figure 2**).

EVs were labeled with anti-CTLA4-APC, anti-FASL-PE, anti-BTLA-BUV395, anti-PDL2-BV650, anti-PDL1-BV786, anti-PD1-BV421, anti-CD39-BV510, anti-CD73-BB515, anti-CD4-BUV395, anti-CD8-BV786, anti-CD3-BUV737, anti-CD19-AF700, anti-CD14-BV650, anti-CD41a-APC-H7, anti-OX40L-BV421 (BD Biosciences), anti-LAG3-PerCP-Cy5.5, anti-TIM3-BV605, anti-TGFβ-PE-Cy7, anti-CD80-BV605 (BioLegend, San Diego, CA), anti-CD11c-APC and anti-CD14-PerCP-Cy5.5 (Thermo Fisher Scientific, Waltham, MA) antibodies (**Supplementary Table 1**).

EVs were acquired at low speed (200 event/s) and quantified in Trucount tubes (BD Biosciences) as previously described (8, 10–12, 16). Trucount tubes contain a known number of beads. It was, therefore, possible to determine the absolute number of EVs in each sample with the following formula: number of EVs counted x number of Trucount beads in the tube)/number of Trucount beads counted.

### Assay of the binding of CD39^+^ EVs to PBMCs

Assays of the binding of EVs to PBMCs were performed as previously described (12). Six aPC samples were pooled for this assay. EVs were concentrated as described above in the section on EV enrichment. EVs were first stained by incubation with an anti-CD39-BV510 antibody (Biolegend) at 4°C for 30 minutes. They were then washed with filter-sterilized PBS (passage through a filter with 0.1 µm pores) by centrifugation for 1 hour at 100,000 x *g* and 4°C, to eliminate the antibodies that had not bound to EVs. The EVs were resuspended in filter-sterilized PBS (passage through a filter with 0.1 µm pores) and the CD39^+^ EVs present in the EV-enriched preparation were counted by flow cytometry in Trucount tubes (BD Biosciences), as described in the “EV phenotyping” section.

PBMCs were isolated by Ficoll density gradient centrifugation. We cultured 2x10^5^ PBMCs with known numbers of CD39^+^ EVs in 96-well plates for 18 hours. The cultures were set up by adding various amounts of the bulk EV-enriched preparation to the culture medium to obtain ratios of PBMCs to CD39^+^ EVs of 100:1, 10:1, 1:1 and 1:10 (PBMCs: CD39^+^ EVs). We studied the interactions of these EVs with CD4^+^ TLs (CD3^+^CD4^+^), CD8^+^ TLs (CD3^+^CD8^+^), DCs (CD11c^+^), monocytes (CD14^+^) and BLs (CD19^+^), which were identified by incubating PBMCs with anti-CD4-BUV395, anti-CD8-BV786, anti-CD3-BUV737, anti-CD19-AF700, anti-CD14-BV650 and anti-CD11c-APC antibodies (BD Biosciences), respectively, for 30 minutes at 4°C. The cells were then washed with PBS.

The culture medium consisted of RPMI 1640 supplemented with 2 mM L-glutamine, 100 μg/mL penicillin/streptomycin, MEM non-essential amino acids (1X), 1 mM sodium pyruvate (Thermo Fisher Scientific) and 5% FBS (Dutscher, Bernolsheim, France). This sterile medium was passed through a filter with 0.1 µm pores in sterile conditions to remove the EVs present in the FBS. At the end of the culture period, the PBMCs were harvested for flow cytometry to assess their co-expression of CD39 originating from EVs (BV510-labeled).

### Assay of the expression of CD39 on BLs after binding to CD39^+^ EVs

We focused on BLs, minimizing the uptake of CD39^+^ EV by other cell types by enriching PBMC preparations in CD19^+^ BLs with a commercial anti-human CD19 particle - DM kit (BD Biosciences), as previously described (10–12). A portion of the BL-rich suspension was used to assess the degree of enrichment by flow cytometry. The cells were labeled by incubation with an anti-CD19-AF700 (BD Biosciences) antibody for 30 minutes at 4°C. BLs and EVs were prepared separately before coculture to investigate their interaction. BLs were labeled with an anti-CD39-BUV737 antibody, and the EV-enriched preparation was labeled with an anti-CD39-BV510 antibody, to make it possible to distinguish cellular CD39 from the CD39 expressed by EVs. For studies of interaction with the CD39 on EVs, EV-enriched preparations from PCs were also labeled with an anti-CTLA4-APC, anti-FASL-PE, anti-BTLA-BUV395, anti-PDL2-BV650, anti-PDL1-BV786, anti-PD1-BV421, anti-CD73-BB515, anti-OX40L-BV421, anti-LAG3-PerCP-Cy5. 5, anti-TIM3-BV605, and anti-TGFβ-PE-Cy7 antibodies.

The CD39^+^ EVs present in the EV-enriched preparation were counted in Trucount tubes (BD Biosciences) as described the EV phenotyping section. The stained BLs and EVs were washed with filter-sterilized PBS and placed in culture (37°C, 5% CO_2_) for 18 hours at an optimal ratio of 1:10 (CD19^+^ BLs: CD39^+^ EVs). The culture was harvested in the same sterile medium and passed through a filter with 0.1 µm pores. CD19^+^ BLs were harvested for flow cytometry to assess their co-expression of CD39 from EVs (BV510-labeled) and cellular CD39 (BUV737-labeled).

### CD39^+^ EV sorting by flow cytometry

For functional assays, CD39^+^ EVs were purified by flow cytometry. All the EVs studied were collected from samples taken from PCs used for the treatment of polytransfused patients. Six aPC samples were pooled for CD39^+^ EV sorting. After EV enrichment, as described above, EVs were labeled with anti-CD39-APC antibody (BD Biosciences) and sorted by flow cytometry (Astrios, Beckman Coulter, Brea, CA) at the CYBIO platform of the Cochin Institute (https://institutcochin.fr/plateformes-technologiques/cybio), as previously described (10–12). The cytometer was calibrated with Megamix-Plus FSC and SSC beads (BioCytex) before each use.

### Proliferation of CD4^+^ TLs after interaction with sorted CD39^+^ EVs

We investigated the role of CD39^+^ EVs in reactivating a CD4^+^ T vaccine memory response. We assessed CD4^+^ TL proliferation as previously described (10), using PBMCs from healthy donors (HDs) vaccinated for tuberculosis. EVs were isolated from PCs, and CD39^+^ EVs were purified by flow cytometry.

PBMCs were isolated by Ficoll density gradient centrifugation, labeled with CFSE (carboxyfluorescein succinimidyl ester) at a concentration of 0.3 µM and incubated at 37°C for 10 minutes. The reaction was stopped by adding 2 mL FBS and incubating the mixture for 1 minute at room temperature. CFSE-labeled cells were cultured in 48-well plates with or without EVs, at a ratio of 1:10 (PBMCs: CD39^+^ EVs). CFSE-labeled cells were also either left unstimulated or were stimulated with *Mycobacterium tuberculosis* purified protein derivative (PPD, Statens Serum Institut, Copenhagen). After incubation for five days at 37°C, under an atmosphere containing 5% CO_2_, cells were stained by incubation with anti-CD4-BUV395 and anti-CD3-BUV737 (BD Biosciences) antibodies for 30 minutes at 4°C. The culture was harvested in the same sterile medium and passed through a filter with 0.1 µm pores. The PBMCs were harvested for flow cytometry to assess lymphoproliferation. Cell division was assessed by timed acquisition flow cytometry analysis for CFSE^lo^ CD3^+^CD4^+^ cells that had and had not interacted with CD39^+^ EVs.

### Immunoglobulin response of BLs after interaction with sorted CD39^+^ EVs

We cultured purified CD39^+^ EVs and purified BLs (CD19^+^) together for four days and then collected the supernatant for an analysis of antibody production. The BLs were prepared as described above in the “Assay of the expression of CD39 on BLs after binding to CD39^+^ EVs” section. EVs were isolated from PCs and CD39^+^ EVs were purified by flow cytometry. For each pool of EVs, several treatments were included in which BLs were left unstimulated or were stimulated with or without EVs. For BL stimulation, we added CD40L (50 ng/mL, R&D Systems, Minneapolis, MN) and CpG (6 μg/mL, ODN 2006, InvivoGen, San Diego, CA) to the culture medium to mimic the action of CD4^+^ TLs. After incubation for 4 days at 37°C under an atmosphere containing 5% CO_2_, the supernatant was recovered and frozen at -80°C. Antibody levels were determined with Luminex technology and a commercial kit (ProcartaPlex Human Antibody Isotyping Panel 7-Plex, Thermo Fisher Scientific) as previously described (10). Antibody concentrations were determined on a MAGPIX device (Luminex, Austin, Texas, USA), with ProcartaPlex software for analysis of the results (Thermo Fisher Scientific).

### Flow cytometry analysis

For EV phenotyping, interaction and functional assays, fluorescence was assessed on an LSR Fortessa flow cytometer (BD Biosciences). The flow cytometry data were analyzed with FlowJo software (v.10.8.1, FlowJo, Ashland, OR).

### Statistical analysis

All analyses were performed with Prism 6.07 software (GraphPad Software, La Jolla, CA). Only significant differences between groups (*P*<0.05) are indicated on the data plots. Details of the statistical tests performed are provided in the legend to each figure. Co-expression was analyzed and plotted with SPICE software version 6.1 (https://niaid.github.io/spice).

## Results

### Characterization of CD39^+^ EVs from platelet concentrates

**Figure 1:**
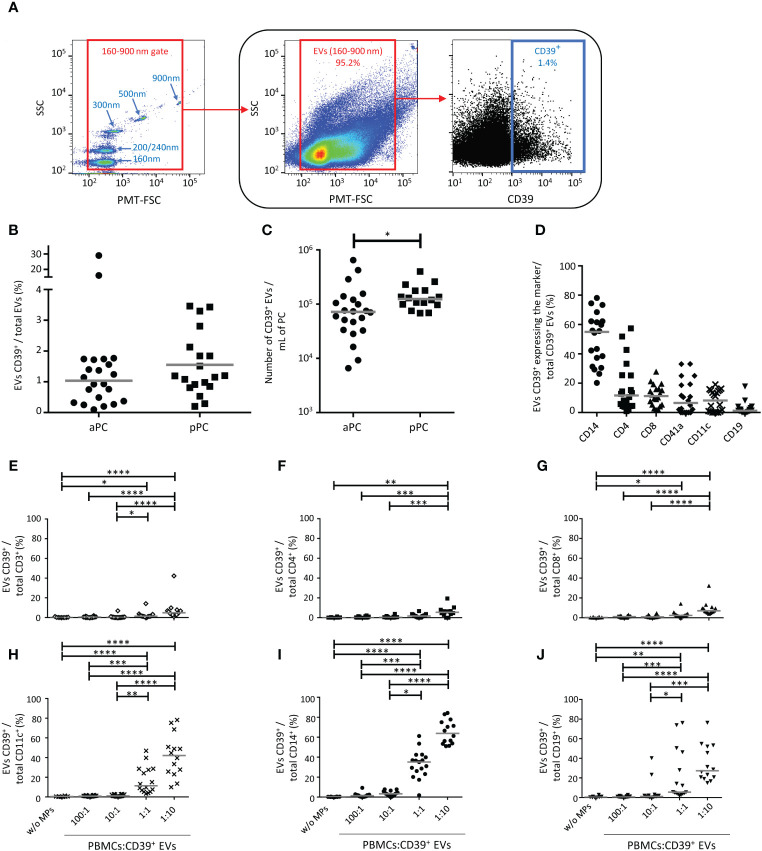
Characterization of CD39^+^ extracellular vesicles (EVs) from platelet concentrates (PCs). **(A)** Example of the gating strategy used in the phenotyping of EVs from healthy donors (HDs) by flow cytometry. On the left, a dot plot showing the settings, based on Megamix fluorescent beads, for the differentiation of particle sizes with diameters of 160 nm, 200/240 nm, 300 nm, 500 nm and 900 nm. Megamix beads were used to define the gate for the EVs studied (on the middle). On the right, an example of the phenotyping of EVs to identify CD39^+^ EVs in the EV gate. **(B)** Percentage of CD39^+^ EVs in apheresis platelet concentrates (aPC) (*n* =20) and pooled platelet concentrates (pPC) (*n* =19). **(C)** Absolute number of CD39^+^ EVs/mL for aPCs (*n* =22) and pPCs (*n* =18). The *P* value was obtained in a Mann-Whitney test: *P < 0.05. **(D)** Expression of cellular origin markers by CD39^+^ EVs isolated from aPCs (*n* =20). The cellular origin of EVs was determined from the presence of cell-specific membrane markers: CD14 (monocytes), CD11c [dendritic cells (DCs)], CD41a (platelets), CD19 [B lymphocytes (BLs)], CD4 and CD8 [T lymphocytes (TLs)]. The horizontal line represents the median. Results are expressed as a percentage of all the EVs for the cells of origin studied. **(E-J)** Interaction of CD39^+^ EVs with immune cells. Percentage of CD39^+^ EVs bound to TLs (CD3^+^, CD4^+^, CD8^+^, **E-G**, respectively), DCs (CD11c^+^, **H**), monocytes (CD14^+^, **I**) and BLs (CD19^+^, **J**) after overnight culture at the following ratios, 1:0, 100:1, 10:1, 1:1 1:10 (PBMCs: CD39^+^ EVs). *n* (1:0, without EVs) =8, *n* (100:1) =16, *n* (10:1) =16, *n* (1:1) =17, *n* (1:10) =14. The horizontal line represents the median. Differences were evaluated by ANOVA. *P* values were obtained in Kruskal-Wallis tests: ****P < 0.0001, ***P < 0.001, **P < 0.01, *P < 0.05.

CD39 expression on EVs (BV510 staining) from PCs was characterized by flow cytometry (**Figure 1A**). The frequency of CD39 expression on EVs isolated from platelet concentrates was variable, with CD39 detected on a mean of 0.95 ± 0.59% aPCs and 1.5 ± 1.0% pPCs (**Figure 1B**). The absolute number of CD39^+^ EVs was 1.19x10^5^ ± 1.52x10^5^ per mL of aPCs and was 1.49x10^5^ ± 8.64x10^4^ per mL of pPCs (**Figure 1C**).

The co-expression of a marker of cellular origin and CD39 on EVs was also assessed by flow cytometry (**Supplementary Figure 3**). We found that 50.3 ± 17.6% of the CD39^+^ EVs co-expressed CD14, 17.2 ± 16.9% co-expressed CD4, 11.7 ± 7.1 co-expressed CD8, 10.4 ± 11.4% co-expressed CD41a, 8.0 ± 7.1% co-expressed CD11c and 2.5 ± 4.1% co-expressed CD19 (**Figure 1D**).

### Immune interactions with CD39^+^ EVs from platelet concentrates

We then cultured PBMCs with CD39^+^ EVs from PCs to evaluate the interaction between these EVs and immune cells: TLs (CD4^+^ and CD8^+^ TLs), BLs, DCs, and monocytes. The cultures were set up with different ratios of cells to CD39^+^ EVs (cells: EVs), to mimic the variation of CD39^+^ EV levels in transfused products (11).

For all cell types studied, the percent interaction between cells and CD39^+^ EVs was significant at a ratio of 1:10 (PBMC: CD39^+^ EVs) in culture (**Figures 1E–J**), corresponding to the maximum number of EVs received during polytransfusions (12). Monocytes were the principal cells that bound CD39^+^ EVs (65.2 ± 11.9% CD14^+^ cells) (**Figure 1I**). DCs bound fewer CD39^+^ EVs than monocytes, with 42.0 ± 21.6% of cells presenting interaction (**Figure 1H**). BLs bound fewer CD39^+^ EVs than DCs (34.5 ± 18.6% of BLs at a 1:10 ratio) (**Figure 1J**). For TLs and a ratio of 1:10, 7.6 ± 10% of CD3^+^ cells (**Figure 1E**), 9.3 ± 7.1% of CD8^+^ TLs ((**Figure 1G**), and 6.0 ± 4.8% of CD4^+^ TLs bound CD39^+^ EVs (**Figure 1F**).

### Phenotypic transfers involving CD39^+^ EVs from platelet concentrates

Given that alloimmunization is linked to alloantibodies and CD39^+^ EVs can interact with BLs, we investigated the functional consequences of CD39^+^ EV interactions with BLs.

**Figure 2:**
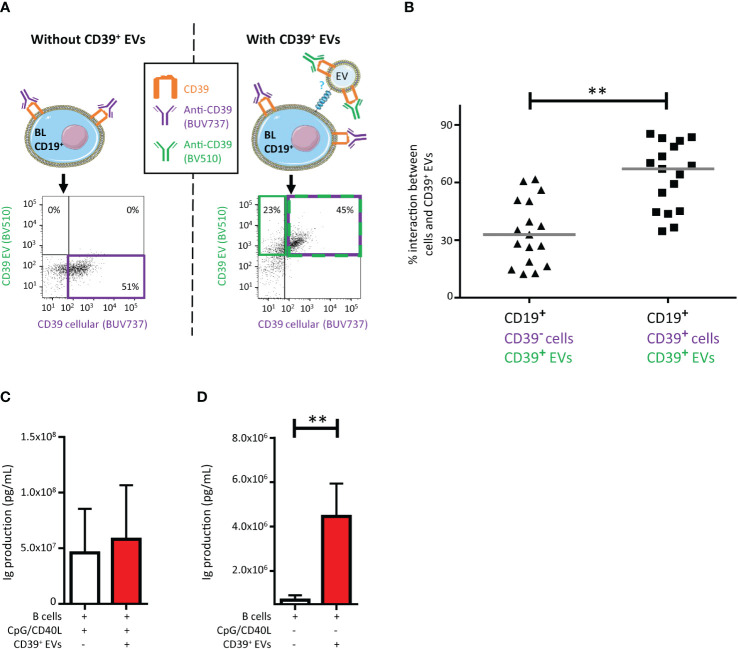
Impact on CD39^+^ B lymphocytes (BLs) of the interaction with CD39^+^ extracellular vesicles (EVs). **(A)** Schematic representation of the assay and example of the gating strategy used to determine the origin of CD39 expression on CD19^+^ BLs from healthy donors (HDs). Cultures were assessed after 18 hours. Before culture, EVs were stained with an anti-CD39-BV510 antibody and BLs were stained with an anti-CD39-BUV737 antibody. Cultures were set up at a ratio of 1:10 (BLs: CD39^+^ EVs). The dot plots presented were generated from gating on CD19^+^ BLs. **(B)** Percentage of CD39^+^ EVs interacting with BLs with and without cellular CD39 expression (*n* =17, five HDs, in five independent experiments). *P* values were obtained in a Wilcoxon test: **P < 0.01. **(C)** Immune activation due to the interaction of CD39^+^ EVs with BLs, with CpG/CD40L stimulation, with or without CD39^+^ EVs at a ratio of 1:10 (BLs: sorted CD39^+^ EVs). Total amount of antibodies secreted per 100,000 BLs after 4 days of culture. Antibody secretion was determined in a Luminex assay. *n* =5, with two HDs in two independent experiments. **(D)** Immune activation due to the interaction of CD39^+^ EVs with BLs, without CpG/CD40L stimulation, with or without CD39^+^ EVs at a ratio of 1:10 (BLs: sorted CD39^+^ EVs). Total amount of antibodies secreted per 100,000 BLs after 4 days of culture. Antibody secretion was determined in a Luminex assay. *n* =5, with two HDs in two independent experiments. *P* values were obtained in a Mann-Whitney test: **P < 0.01.

We investigated the modulation of the CD39 phenotype of BLs by EVs in culture at a 1:10 ratio (BLs: CD39^+^ EVs). CD39 acquisition was assessed by flow cytometry. We differentiated the CD39 originating from EVs from that originating from the cells by labeling cells and EVs with different colored antibodies against CD39 and then washing them before culture (12). The CD39 BUV737 antibody was used for BL staining and the CD39 BV510 antibody was used for EV staining (**Figure 2A**). The BLs that interact with EVs may or may not already express CD39 (those that do are stained with BUV737). We found that CD39^+^ EVs interacted significantly more frequently with CD39^+^ BLs (67.1 ± 17.3%) than with CD39^-^ BLs (32.9 ± 16.9%, *P*<0.01) (**Figure 2B**).

We then investigated the impact of purified CD39^+^ EVs on BL function by assessing immunoglobulin production. After the polyfunctional stimulation of BLs, no modification of immunoglobulin secretion (Ig) was observed when these cells were cultured with CD39^+^ EVs (**Figure 2C**), regardless of the Ig subclass considered (**Supplementary Figure 4**). However, CD39^+^ EVs alone were able to activate Ig production by BLs (*P <*0.01) (**Figure 2D**), also regardless of the Ig subclass considered (**Supplementary Figure 4**).

**Figure 3:**
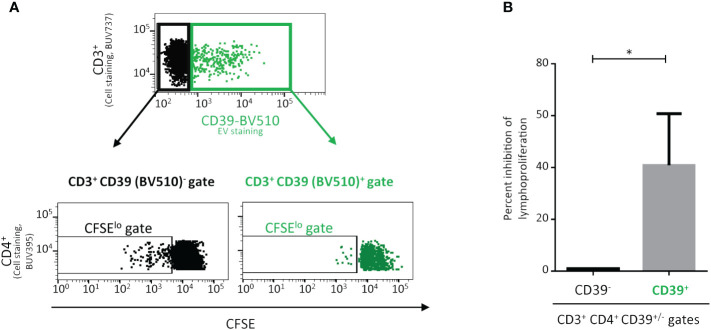
Impact on the proliferation of CD4^+^ T-lymphocytes (TLs) of the interaction with CD39^+^ extracellular vesicles (EVs). Proliferation of CD4^+^ T cells cultured with CD39^+^ EVs labeled with an anti-CD39-BV510 antibody. PMBCs were obtained from healthy donors (HDs) vaccinated against tuberculosis. PMBCs were incubated for five days with *Mycobacterium tuberculosis* purified protein derivative (PPD) and CD39^+^ EVs (BV510) at a ratio of 1:10 (PBMCs: CD39^+^ EVs). **(A)** Lymphoproliferation was assessed for the CD39 BV510^neg^ CD4^+^ TL population and the CD39 BV510^pos^ CD4^+^ TL population. The upper representation shows only the CD3^+^ lymphocyte gate, the data for CD3^-^ lymphocytes are not displayed. The lower representation shows the carboxyfluorescein succinimidyl ester (CFSE) signal only for the CD4^+^ gate, data for CD4^-^ cells are not displayed. Cell division was assessed with the CFSE^lo^ TL subpopulation (the data shown were obtained in a single experiment representative of five experiments performed, with one donor per experiment). **(B)** Effect of CD39^+^ EVs on CD3^+^CD4^+^ TL proliferation (*n*=5, in five independent experiments). The percent inhibition of lymphoproliferation with CD39^+^ EVs was calculated and normalized, taking the lymphoproliferation of CD3^+^CD4^+^CD39^neg^ cells as the maximum proliferation for each experiment. *P* values were obtained in Mann-Whitney tests: *P < 0.05.

As alloimmunization and alloantibody production are totally dependent on CD4^+^ TLs (33), and despite the limited interaction of CD39^+^ EVs with CD4^+^ TLs (**Figure 1F**), we studied the impact of these CD39^+^ EVs on CD4^+^ T lymphoproliferation (**Figure 3A**). The interaction of CD39^+^ EVs with CD4^+^ TLs did not completely abolish CD4^+^ T lymphoproliferation after antigenic restimulation (**Figure 3A**). Lymphoproliferation halted after one cycle of cell division (**Figure 3A**). We therefore observed a 40.9 ± 19.3% inhibition of CD4^+^ T-cell proliferation after antigenic restimulation (**Figure 3B**).

### Distribution of immune molecules on CD39^+^ EVs

The phenotype of EVs is linked to cell budding and is, therefore, heterogeneous, depending on the immune molecules present on the surface of the cell of origin (10). EVs can be immunoregulatory or immunostimulatory (10). Our unexpected results with BLs may therefore be related to the phenotype of CD39^+^ EVs (**Figure 2D**). We tested this hypothesis and investigated whether the binding of CD39^+^ EVs to BLs was random or involved receptor-ligand binding, by phenotyping the CD39^+^ EVs for the co-expression of other immunomodulatory molecules.

**Figure 4:**
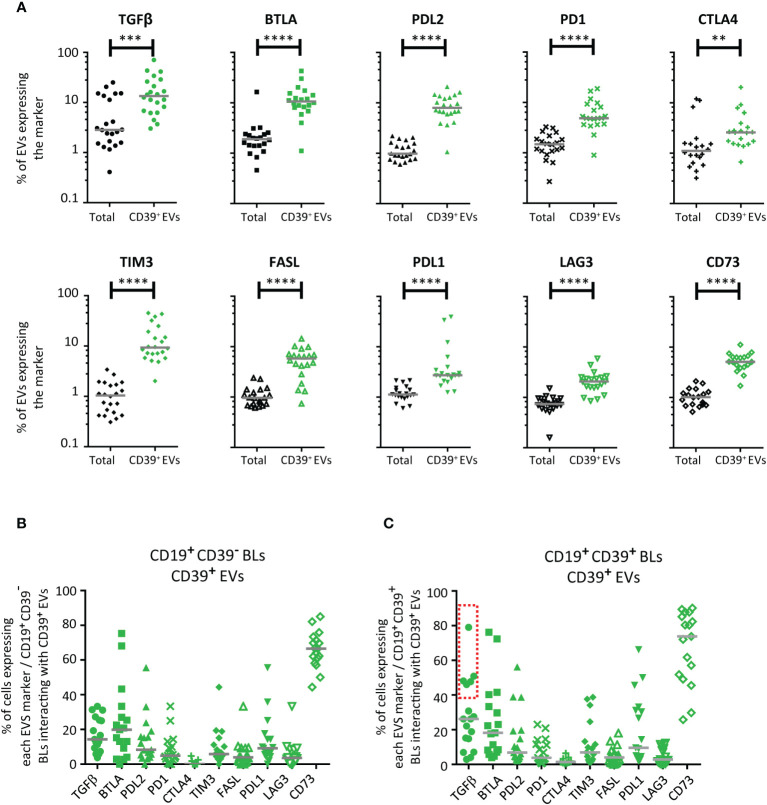
Distribution of immunomodulatory molecules on CD39^+^ EVs. **(A)** Distribution of 10 immunoregulatory molecules on total EVs (black) and on CD39^+^ EVs (green) (*n* =19 except for CD73 with *n* =17, five independent experiments). For total EVs, results are expressed as a percentage of labeled EVs for all EVs studied. For CD39^+^ EVs, results are expressed as a percentage of labeled EVs for CD39^+^ EVs only. **(B, C)** Co-expression of immunomodulatory molecules on CD39^-^ **(B)** and CD39^+^ **(C)** BLs after interaction with labeled CD39^+^ EVs. BLs were labeled with an anti-CD39-BUV737 antibody to make it possible to distinguish cellular CD39 from the CD39 expressed by EVs (anti-CD39-BV510 antibody-labeled). We prevented the uptake of CD39^+^ EVs by other cell types, by enriching the PBMC preparation in CD19^+^ BLs with a commercial anti-human CD19 particle - DM kit (BD Biosciences), as previously described (10–12). For studies of interactions with the immunoregulatory molecules present on CD39^+^ EVs, EV preparation from PCs were also labeled with anti-CTLA4-APC, anti-FASL-PE, anti-BTLA-BUV395, anti-PDL2-BV650, anti-PDL1-BV786, anti-PD1-BV421, anti-CD73-BB515, anti-LAG3-PerCP-Cy5.5, anti-TIM3-BV605, and anti-TGFβ-PE-Cy7 antibodies. The co-expression of vesicular CD39 and 10 vesicular immunoregulatory molecules on LBs was assessed by cytometry after culture (*n* =17, except for *n* =9 for CTLA4, five independent experiments). The BLs: CD39^+^ EVs culture ratio used was 1:10. The horizontal line represents the median. *P* values were obtained in a Mann-Whitney test: ****P< 0.0001, ***P< 0.001, **P < 0.01.

We assessed the expression of CTLA4, PD1, PDL1, PDL2, BTLA, FASL, TIM3, LAG3, TGFβ and CD73 on CD39^+^ EVs and, for the purposes of comparison, on all EVs (**Supplementary Figure 5**). In the analysis of all EVs considered together, TGFβ was detected on 2.9 ± 7.6%, BTLA was detected on 1.9 ± 3.4% and CTLA4, FASL, PD1, TIM3, PDL2, CD73, LAG3 and PDL1 were each detected on less than 1.5% of EVs (**Figure 4A**).

Co-expression was significantly more frequent on CD39^+^ EVs than on total EVs. For CD39^+^ EVs, TGFβ was also detected on 12.0 ± 17.3%, BTLA on 10.6 ± 9.5%, TIM3 on 8.7 ± 9.6%, PDL2 on 8.0 ± 4.9%, FASL on 5.8 ± 3.4%, PD1 on 4.9 ± 4.6%, CD73 on 5.1 ± 2.2%, PDL1 on 2.7 ± 10.6%, CTLA4 on 2.6 ± 4.5%, and LAG3 on 2.1 ± 1.2% of CD39^+^ EVs (**Figure 4A**).

We then assessed the expression of all the immune molecules present on EVs on the BLs that had interacted with CD39^+^ EVs. Results were obtained separately for BLs with and without cellular CD39 expression (CD39^+^ and CD39^-^ BLs, as determined by BUV737 staining).

After interaction with CD39^+^ EVs, cellular CD39^-^ BLs and cellular CD39^+^ BLs were found to express all the molecules present on the surface of CD39^+^ EVs (**Figures 4B, C**). However, for TGFβ, the interaction differed between cellular CD39^-^ BLs and cellular CD39^+^ BLs (identified on the basis of BUV737 staining). Two groups of cellular CD39^+^ BLs were observed, one of which (>40%) displayed an interaction for TGFβ (**Figure 4C**); no such interaction was observed for CD39^-^ BLs (**Figure 4B**).

**Figure 5:**
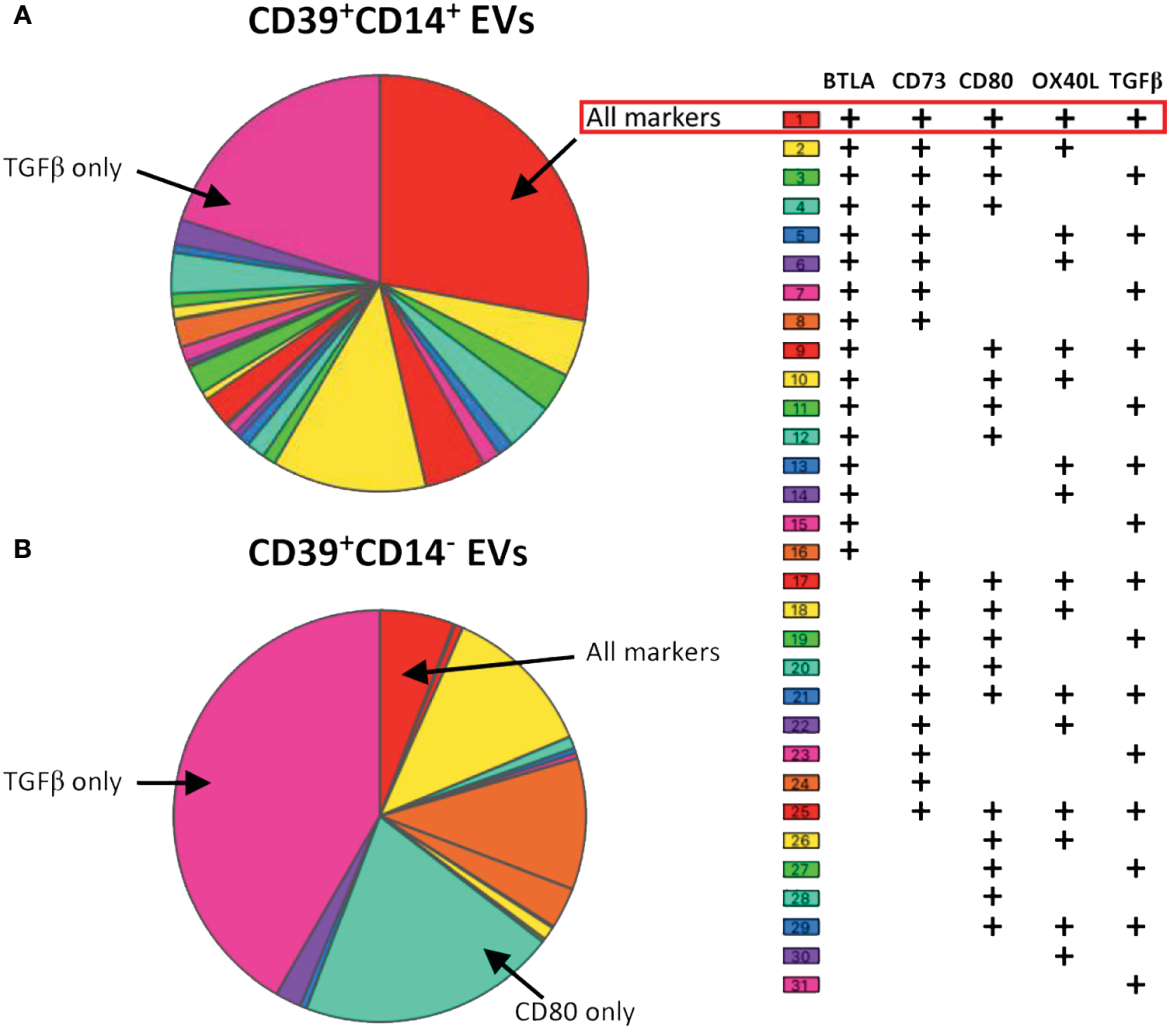
Co-expression of BTLA, CD73, CD80, OX40L and TGFβ on CD39^+^ EVs from aPCs. Co-expression data are presented for EVs with **(A)** or without **(B)** a monocyte origin (CD14^+^ EVs). Co-expression was analyzed, and the data were plotted with SPICE software version 6.1. Co-expression was analyzed for 17 aPCs (three independent experiments).

The relationships between the expression of each of the molecules on EVs and their functions were complex. Indeed, co-expression has been reported (10, 12) and may be associated with the immune cell from which the EVs originated, not only for regulatory molecules, but also for activator molecules. We tested this hypothesis by studying the co-expression of molecules on the surface of monocytes, the cell type of origin for more than 50% of CD39^+^ EVs (**Figure 1D**). We found that the co-expression profiles of the CD39^+^ EVs derived from transfusion products depended on their monocytic or non-monocytic origin (**Figure 5**). We assessed the levels of expression on CD39^+^CD14^+^ EVs and CD39^+^CD14^-^ EVs of the most frequently expressed molecules: BTLA, CD73, CD80, OX40L and TGFβ (**Figure 5**). CD80 and OX40L were selected because they are crucial for immune synapses and, thus, for the activation of TLs and BLs (31).

The profiles obtained differed considerably between CD39^+^ EVs of monocytic and non-monocytic origin. Thirty-one combinations were established for CD39^+^CD14^+^ EVs, and 50% of CD39^+^ EVs expressed either only TGFβ or all the markers studied (**Figure 5**). The phenotype of CD39^+^CD14^-^ EVs was much less extensive, with only 13 combinations found (**Figure 5**). In addition, the proportion of EVs expressing TGFβ was much higher (56%, **Figure 5B**). The same was true for CD80 only, which was more frequently expressed by CD39^+^CD14^-^ EVs, than by CD39^+^CD14^+^ EVs, on which its expression was very weak (11% vs. 2%, respectively, **Figure 5**).

## Discussion

The proportion of CD39^+^ EVs isolated from PCs did not differ between aPC and pPC samples, with a median value close to 1%. The aPC samples displayed some heterogeneity, and the pPC samples were even more heterogeneous. We detected larger numbers of CD39^+^ EVs in pPCs than in aPCs due to the larger total number of EVs in pPCs than in aPCs (**Supplementary Figure 6**).

The total density of EVs was 3.2x10^6^ ± 1.9x10^6^ EVs/mL for aPCs and 1.0x10^6^ ± 6.9x10^6^ EVs/mL for pPCs. Based on these findings, we would expect 6.3x10^8^ EVs for aPCs and 2.0x10^9^ EVs for pPCs to be introduced into the patient during transfusion. Moreover, up to 9% of donors provided aPCs with a very high level of CD39^+^ EVs (10 times higher than that for other donors) with a mean value of 10.7x10^7^ CD39^+^ EVs/platelet concentrate. Variations of the number of EVs have a major effect on the immunomodulatory function of EVs (10–12, 15, 16, 34). Furthermore, the use of PCs in which more than 30% of the EVs present express CD39 may have a significant effect on the immunity of the transfused patient.

The heterogeneity of EVs in PCs varied between samples, consistent with published findings (8, 11, 12). The number of EVs in transfusion products is highly variable and depends, above all, on the physiological state of the donor (8, 10, 35–40), the method used to prepare platelet concentrates (41–43) and the cellular origin of the EVs. Indeed, PCs have been reported to contain a large proportion of monocyte EVs (MEVs) (8) and MEV levels were also high in the samples studied here (**Supplementary Figure 7**). This finding is of particular importance because monocytes display high levels of CD39 expression (44). Our data confirm that the CD39^+^ EVs were generated principally by the budding of monocytes.

The functional effects of EVs, including immunoregulation in particular, are linked to their interactions with cells (12, 13, 45, 46). Given the diverse cellular origins of CD39^+^ EVs, we studied their interactions with several different types of immune cells (TLs, BLs, DCs, monocytes). All CD39^+^ EVs were able to interact with these cells and interactions with all populations were stronger for higher proportions of CD39^+^ EVs in culture (**Figures 1E–J**). However, the interaction with these EVs differed between the cell types studied, the most marked difference being that between effector cells and antigen-presenting cells. These differences may be explained by the intrinsic properties of some of these cells (internalization/phagocytosis). However, this internalization of EVs by antigen-presenting cells is not sufficient in itself to explain all observations. Other EVs, such as CD27^+^ EVs, interact particularly well with CD4^+^ TLs (12), as observed in our previous studies with other EVs of lymphocytic origin (data not shown), consistent with the involvement of ligand/receptor pairs present on the surface of EVs and immune cells.

Patients with hematologic disorders undergo polytransfusion, which may lead to alloimmunization against HLA-I, but 80% of patients undergoing multiple transfusions never develop this condition. This difference in the immunological profiles of patients undergoing transfusion is highly informative for the immunological management of patients, particularly for immunotherapies. We therefore focused on the interactions of EVs with BLs, which produce the anti-HLA class I antibodies principally responsible for alloimmunization.

Our interaction tests revealed that the exchanges between EVs and cells were not completely random, as interactions occurred preferentially with BLs that already expressed CD39. Indeed, as recently reported, these interactions resulted in part from ligand-receptor binding (12). However, CD39 is an ectoenzyme with no known membrane ligand. Nevertheless, EVs display several other immunoregulatory molecules on their surface that can be co-expressed on CD39^+^ EVs (10), as confirmed by our findings. These other immunoregulatory molecules were more strongly expressed on EVs that expressed CD39 than in the total EV population. The interactions of CD39^+^ EVs with BLs already expressing CD39 seemed to involve some immunoregulatory molecules more frequently than others. Indeed, the CD39^+^ EVs that interacted with CD39^+^ BLs also frequently expressed TGFβ (>40%). This is hardly surprising because TGF-β has been shown to be particularly likely to induce the expansion of CD39^+^ Tregs (47). Moreover, like the other immune checkpoints studied (BTLA, CTLA4 and TIM3), TGFβ is expressed on activated TLs and has a major immunoregulatory function.

As the CD39^+^ EVs co-expressed all the immunoregulatory molecules studied, we performed a functional study to assess the impact of these EVs on BLs. Unfortunately, CD39^+^ EVs did not inhibit Ig production after strong polyclonal stimulation (CpG+CD40L) in culture. Surprisingly, whatever the Ig subclass (IgM, IgG or IgA) considered, the CD39^+^ EVs increased antibody production by BLs. However, total levels of Ig production were much lower than observed with full polyclonal stimulation. We have already described this effect on humoral immunity *in vitro* and in a model in which mice were transfused with heterologous EVs *in vivo* (10). The exact mechanism involved remains unknown, but the heterogeneity of EVs may account for these unexpected results. CD39 is not expressed exclusively by immunoregulatory cells. Immune regulatory molecules, such as CD39, are expressed primarily by activated immune cells, and some CD39^+^ EVs may also express markers of immune activation (10, 16, 38). For example, the CD40L present on EVs may interact directly with LBs (10, 16). EVs also contain CpG dinucleotides (mitochondrial GpG), which can be detected by TLR9 on BLs (9).

This heterogeneity of immunomodulation molecule expression on the surface of EVs generally leads to the activation of effector or regulatory cells (4, 9–15, 45). We previously showed that TGFβ EVs could activate both Tfh and Treg cells (10). The interaction of CD39^+^ EVs with BLs was complex, with multiple subpopulations of CD39^+^ EVs identified and other immunomodulation molecules implicated. TGFβ, BTLA, CTLA4 and TIM3 were the immunoregulatory molecules most frequently expressed with CD39. One of the limitations of this assay was our focus on CD39^+^ EVs, an immune subpopulation of EVs. As a result, the rates of expression of these molecules on total EVs was necessarily lower than that on CD39^+^ EVs, as the total EV population contains non-immune EVs originating from red blood cells, adipocytes and endothelial cells, for example.

In addition, these molecules confer few or no immunoregulatory properties on CD39^+^ EVs. For full CD39 ectoenzyme functionality, these CD39^+^ EVs would need to co-express CD73 for the production of extracellular adenosine. However, only 5.4% of CD39^+^ EVs also expressed CD73. If we leave the coexpression of molecules to one side and simply add the mean values for the 10 immunoregulatory molecules studied, then these molecules are expressed by only 86.8% of CD39^+^ EVs. This sheds light on the possible importance of other types of co-expression and activating molecules.

Indeed, immune activator molecules, such as CD80 and OX40L, were also expressed. The observed heterogeneity of expression for these molecules was investigated further. TGFβ was the most important of the immunoregulatory molecules expressed by EVs, but immunomodulation molecule profile differed between CD39^+^ EVs originating from monocytes and those originating from other cells. We found that a significant proportion of CD39^+^CD14^-^ EVs also strongly expressed CD80, independently of the other regulatory molecules studied. CD80 may be the vector for the interaction of CD39^+^ EVs with CD28^+^ or CTLA4^+^ cells. In both cases, CD4^+^ T cells are the main target of interest. Thus, the interaction of CD39^+^ EVs with CD4^+^ TLs is responsible for the decrease in lymphoproliferative capacity.

Cooperation between T cells and B cells via costimulatory molecules, such as CD28/CD80 and OX40L/OX40 (also present on CD39^+^ EVs), leads to B-cell activation and differentiation. Thus, our data suggest that these CD39^+^ EVs may have complex functional consequences for BLs, ranging from the inhibition to the activation of antibody production.

EVs are important intercellular communication molecules in physiology and pathophysiology, contributing to phenotypic and functional changes in immune cells (9, 12, 34, 45, 46, 48). The phenotypes of EVs and their proportions in PCs governed their immunomodulatory effects. The presence of high concentration of CD39^+^ EVs in PCs may have major implications for the management of the patients with hematologic disorders and for our understanding of anti-HLA alloimmunization after platelet transfusion. Indeed, leukemic EVs drive the progression of myeloid leukemia by promoting Tregs from dysfunctional subsets of effector T cells with a high level of cellular CD39^+^ expression (49, 50). A supply of transfused CD39^+^ EVs and the cellular interaction observed would therefore reinforce the maintenance of these phenotypes.

Moreover, immunotherapy with antibodies directed against CD39 and CD73 is currently under evaluation for cancer treatment (17). However, competition between EVs and the targets of monoclonal antibodies used for immunotherapy has already been reported (12), and CD39^+^ EVs may also interact with these treatments, potentially decreasing their efficacy.

## Data availability statement

The original contributions presented in the study are included in the article/**Supplementary Materials**, further inquiries can be directed to the corresponding author/s.

## Ethics statement

The studies involving humans were approved by Etablissement Francais du Sang. The studies were conducted in accordance with the local legislation and institutional requirements. The participants provided their written informed consent to participate in this study.

## Author contributions

AD: Formal analysis, Investigation, Visualization, Writing – original draft. AL: Formal analysis, Investigation, Visualization, Writing – original draft. MT: Formal analysis, Methodology, Writing – review & editing. MP: Writing – review & editing. LC: Formal analysis, Investigation, Visualization, Writing – original draft. DN-L: Writing – review & editing. MK: Writing – review & editing. SC: Writing – review & editing, Resources. FP: Writing – review & editing. BV: Conceptualization, Data curation, Formal analysis, Funding acquisition, Investigation, Methodology, Project administration, Supervision, Validation, Writing – original draft, Writing – review & editing.
