# Supplementary material for: Immune interactions and regulation with CD39+ extracellular vesicles from platelet concentrates
Source: Front Immunol. 2024 Jun 14;15:1397967. doi: 10.3389/fimmu.2024.1397967 (PMC11211594; doi:10.3389/fimmu.2024.1397967)
Supplement: Supplementary file 1 [file DataSheet_1.docx]

**Immune interactions and regulation with CD39^+^ extracellular vesicles from platelet concentrates**

Adèle Silane Delorme, Alexandra Laguide, Marie Tamagne, Marion Kléa Pinheiro, Léonie Cagnet, Deborah Neyrinck-Leglantier, Mehdi Khelfa, Sabine Cleophax, France Pirenne and Benoît Vingert.

**Supplemental data**

- Supplemental Table 1. Antibodies for flow cytometry, page 2
- Supplemental Figure 1. Refrigerated centrifugation and lack of effect on platelet budding, page 3
- Supplemental Figure 2. Performance of the flow cytometer for EV detection, page 4
- Supplemental Figure 3. Example of flow cytometric phenotyping for markers of cellular origin on EVs, page 5
- Supplemental Figure 4. Antibody subclasses after the stimulation of BLs with CD39^+^ EVs, page 6
- Supplemental Figure 5. Example of flow cytometric phenotyping for immune system inhibitory molecules on EVs, page 7
- Supplemental Figure 6. Number of EVs present in platelet concentrates, page 8
- Supplemental Figure 7. Expression of markers of cellular origin on EVs isolated from aPCs, page 9

**
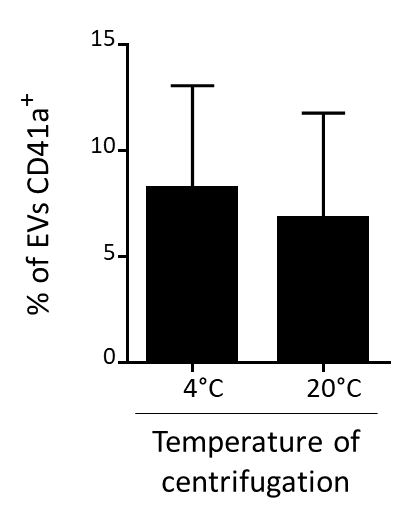
**

**Supplemental Figure 1**. **Centrifugation temperature and lack of effect on platelet budding.** The expression of CD41a was assessed on EVs after the first two differential centrifugations at 4°C or at 20°C. *n*=11 HDs, two independent experiments. No significant difference was found between the two temperatures.


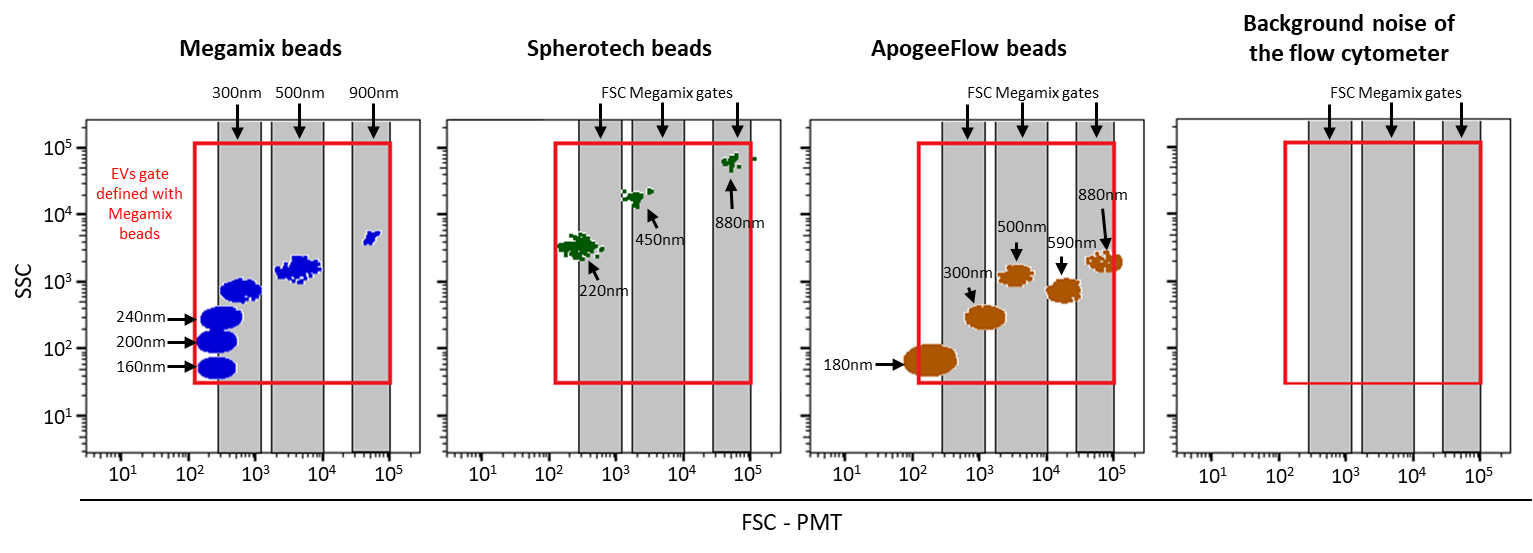


**Supplemental Figure 2.** **Performance of the flow cytometer for EV detection.** Fluorescence was assessed with an LSR Fortessa flow cytometer with a small‐particle option for nanoscale cytometry (BD Biosciences). This option consists of photomultiplier (PMT)‐coupled forward scatter (FSC) detection. The performance of the flow cytometer was checked with three types of calibration beads for flow cytometry: BioCytex (latex beads, Megamix SCC and FSC kits: 160 nm, 200 nm, 240 nm, 300 nm, 500 nm and 900 nm), Spherotech (latex beads, 220 nm, 450 nm and 880 nm) and ApogeeFlow (silica beads, 180 nm, 300 nm, 590 nm, 880 nm and 500 nm fluorescent latex beads). These kits were used to standardize FSC‐PMT parameters for definition of the EV gate and evaluation of the background noise of the machine for EVs.


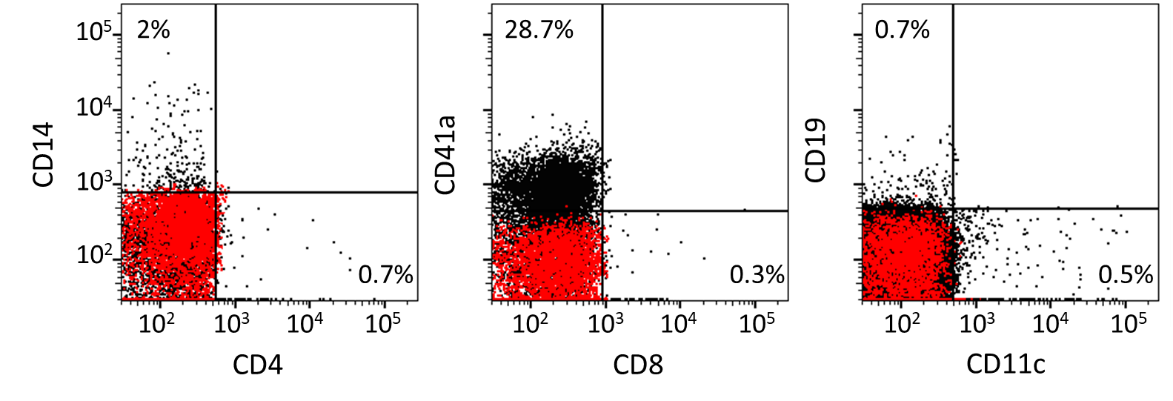


**Supplemental Figure 3. Example of flow cytometric phenotyping for markers of cellular origin on EVs.** The expression of CD14, CD4, CD41a, CD8, CD19 and CD11c on EVs, used to determine the cellular origin of EVs, is shown on this dot plot. Black dots indicate labeling for a marker of cell origin (CD14, CD4, CD41a, CD8, CD19 and CD11c) and red dots indicate isotypic labeling. The results are expressed as a percentage of the total EV population for each of the cell subpopulations considered.

**
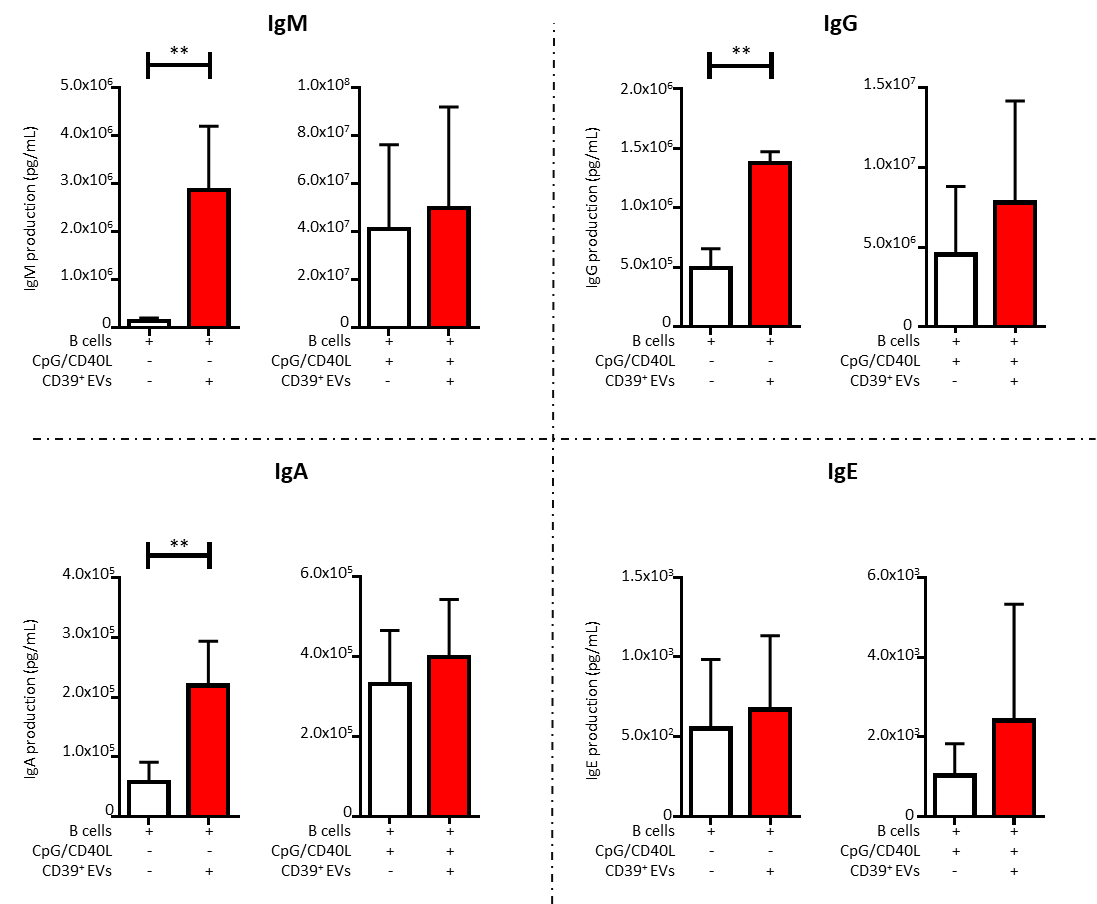
**

**Supplemental Figure 4. Antibody subclasses after the stimulation of BLs with CD39^+^ EVs.** Antibody secretion is reported for 100,000 BLs after 4 days in culture at a ratio of 1:10 (BLs: sorted CD39^+^ EVs) with or without CpG/CD40L stimulation. Ig subclasses were determined in Luminex assays. *n*=5, two independent experiments. *P* values were obtained in a Mann-Whitney test: ** *P*<0.01. Only significant differences between groups (*P*<0.05) are indicated on the data plots.

**
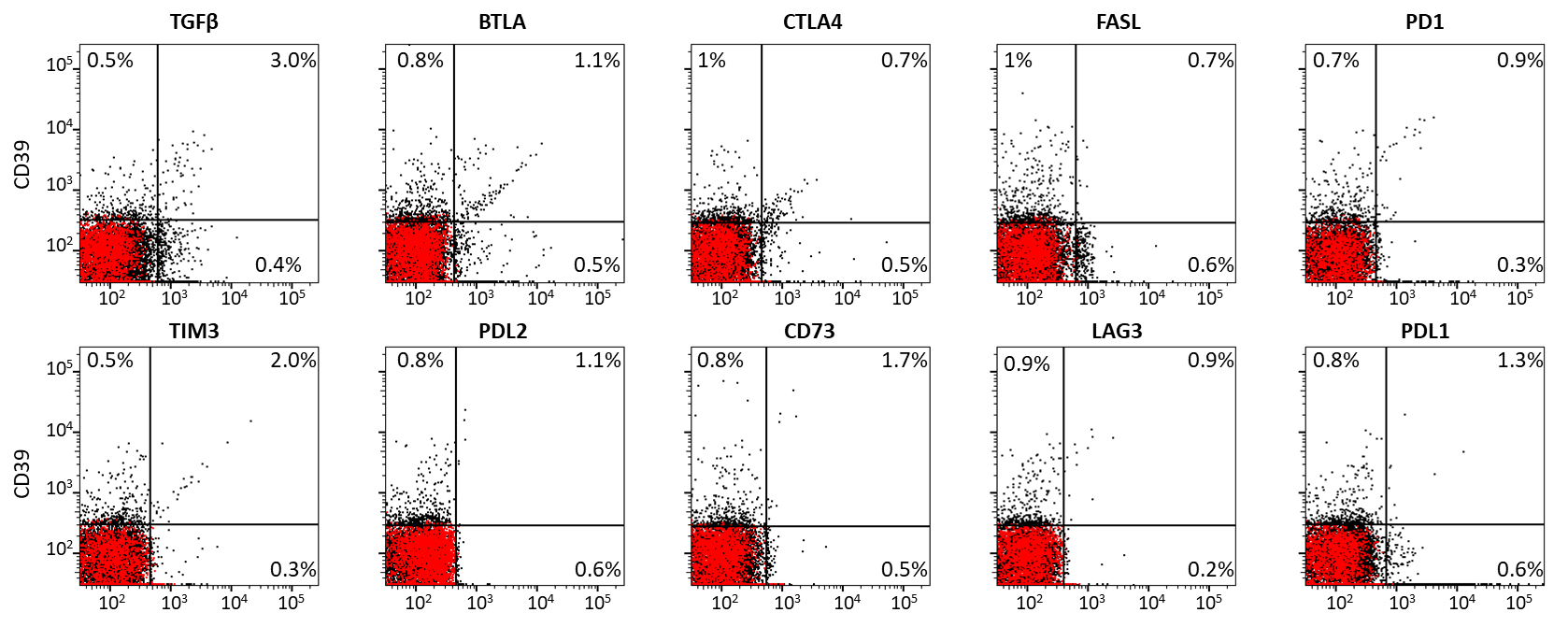
Supplemental Figure 5. Example of flow cytometric phenotyping for immune system inhibitory molecules on EVs.** Co-expression of immune system inhibitory molecules (TGFβ, BTLA, CTLA4, FASL, PD1, TIM3, PDL2, CD73, LAG3, PDL1), on the *x* axis, with CD39 expression of EVs, on the *y* axis. Labeling is presented as a dot plot with black dots for labeling with immunoregulatory antibodies (TGFβ, BTLA, CTLA4, FASL, PD1, TIM3, PDL2, CD73, LAG3 or PDL1) and red dots for isotypic labeling. The results are expressed as a percentage of the total EV population.


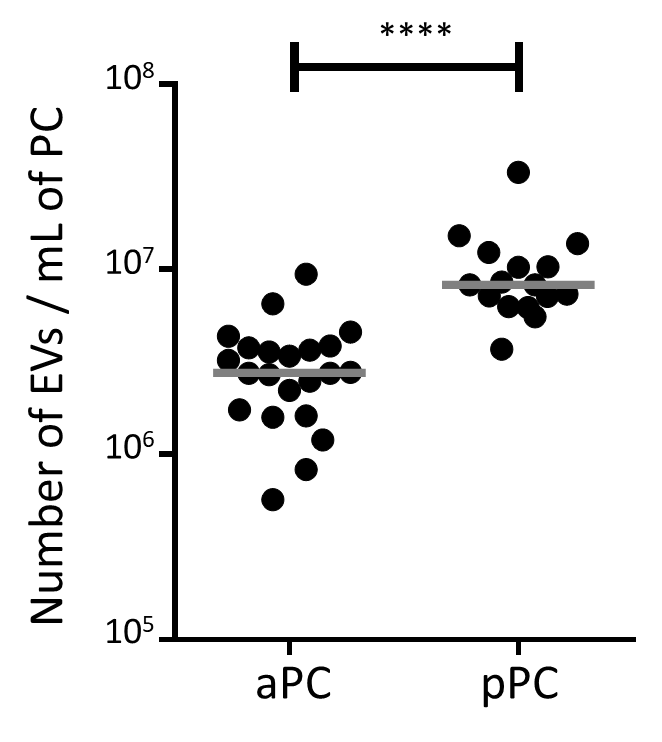


**Supplemental Figure 6**. **Number of EVs present in platelet concentrates.** Results are presented for *n*=22 aPCs (in five independent experiments) and *n*=18 pPCs (in one experiment). *P* values were obtained in Mann-Whitney tests: **** *P*<0.0001.

**
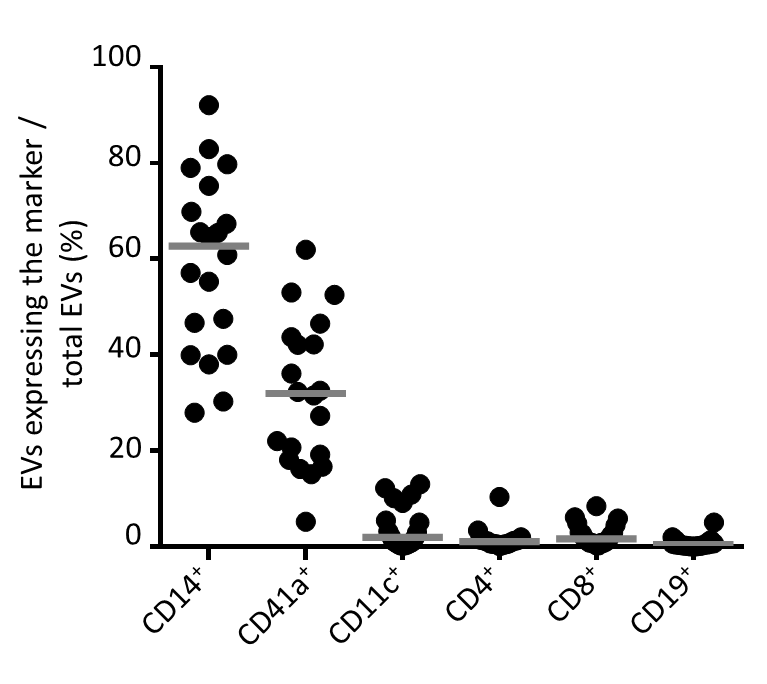
**

**Supplemental Figure 7. Expression of markers of cellular origin on EVs isolated from aPCs.** The cellular origin of EVs was determined on the basis of the presence of cell-specific membrane markers: CD14 (monocytes), CD11c (DCs), CD41a (platelets), CD19 (B cells), CD4 and CD8 (T cells) (*n*=20). The horizontal line represents the median. Only significant differences between groups (*P*<0.05) are indicated on the data plots. The results are presented as a percentage of total EVs for each cellular origin considered.
